# Gas-Phase Conversion of 1,3-Dithiolane-2-Thione Into 1,3-Dithiolan-2-One Over Molybdenum Trioxide

**DOI:** 10.3389/fchem.2019.00204

**Published:** 2019-04-05

**Authors:** R. Alan Aitken, Thomasine E. Curzon, Matthew J. Andrews

**Affiliations:** EaStCHEM School of Chemistry, University of St Andrews, St Andrews, United Kingdom

**Keywords:** oxidation, gas-phase reaction, thione, flow chemistry, green chemistry

## Abstract

Gas-phase reaction of 1,3-dithiolane-2-thione over molybdenum trioxide supported on pumice stone results in efficient conversion into 1,3-dithiolan-2-one. The solid reagent is regenerated on exposure to air and thus acts as a catalyst for the overall conversion of the thione and oxygen from the air into the ketone and sulfur dioxide. The process can be carried out under either dynamic vacuum or atmospheric pressure flow conditions and using a solid reagent prepared either by physical mixing of MoO_3_ with the support or by solution impregnation, with an isolated yield of up to 67% obtained.

## Introduction

**Scheme 1:**
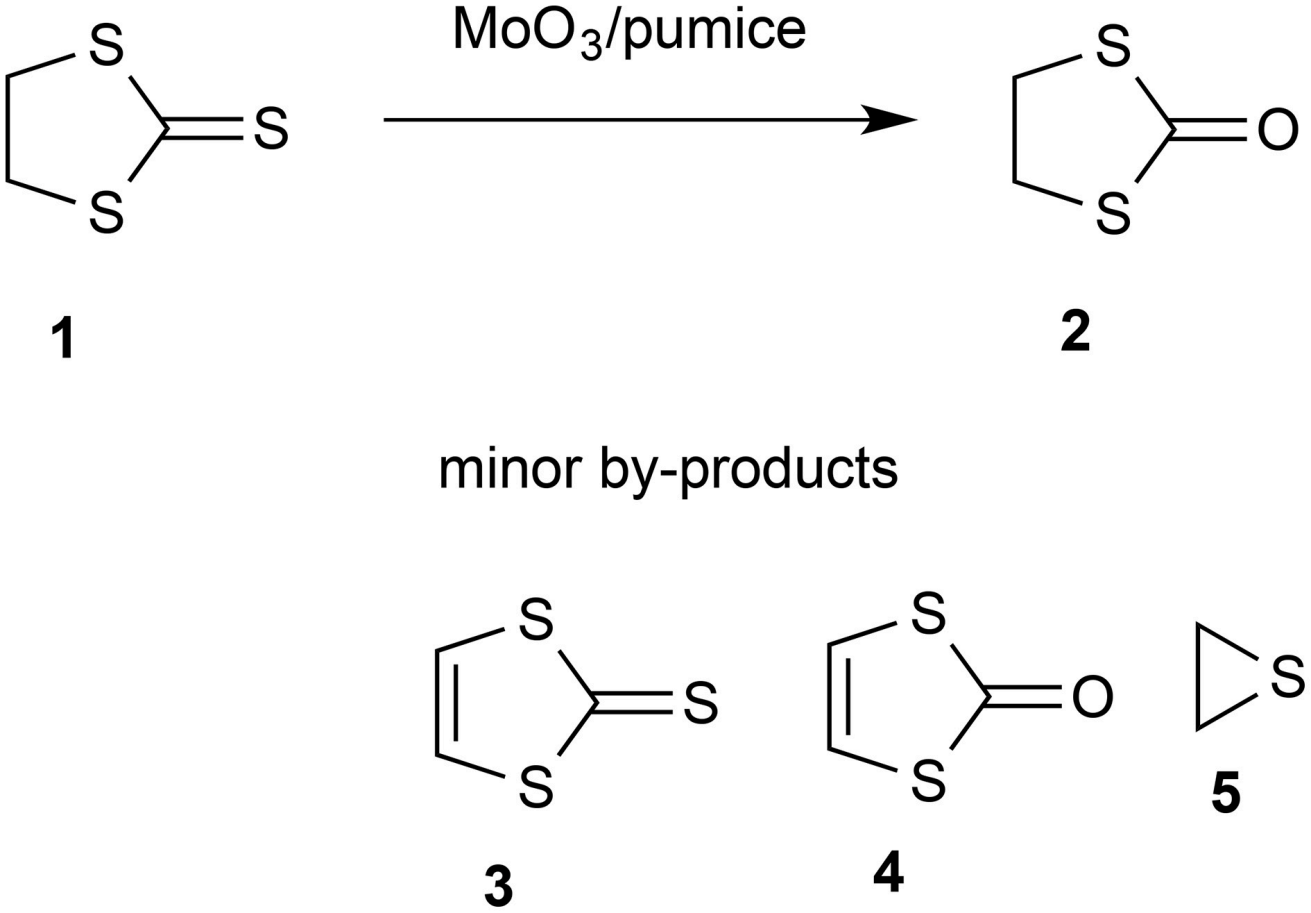
Gas-phase conversion of **1** into **2** with minor by-products also identified.

There has been considerable recent interest in the use of flash vacuum pyrolysis (FVP) for the synthesis of heterocyclic compounds (Aitken and Boubalouta, 2015) and there have been occasional reports of its use in synthesis of pharmaceutical agents (Horn and Gervay-Hague, 2009) and natural products (Harras et al., 2017). The inherently clean conditions where each substrate molecule reacts during a brief “contact time” in the hot zone in the absence of solvents, reagents, or products and the products are then rapidly condensed in a cold trap at cryogenic temperatures can lead to high yields of otherwise inaccessible products. An extra dimension to the normal FVP through an empty tube is added by using a tube packed with a solid reagent or catalyst and such “vacuum gas-solid reactions” (Denis and Gaumont, 1997) have been successful in various areas. Among these is the use of solid bases such as potassium t-butoxide, potassium hydroxide, and sodium carbonate to bring about elimination reactions (Guillemin and Denis, 1988; Guillemin et al., 1988; Aitken et al., 1999) and metals such as magnesium (Aitken et al., 1997a,b, 2002, 2017; Aitken and Oyewale, 2015), zinc (Chapman et al., 1976; Rozsondai et al., 1989) and silver (Binnewies et al., 1984) to bring about dehalogenation. Solid catalysts such as zeolites and barium tungstate can also profoundly influence the product distribution in gas-phase processes involving reactive intermediates (Moyano et al., 2007, 2010; Lener et al., 2013). Attracted by a brief thesis report that FVP over molybdenum trioxide was effective in dehydrogenating tetrahydroquinolines and -isoquinolines to the fully aromatic heterocycles (McDougald and McNab, 2000), we have shown that this system is effective in aromatizing a range of partly hydrogenated heterocycles. In the course of this study we examined 1,3-dithiolane-2-thione **1**. Preliminary experiments established that there was no significant dehydrogenation but instead exchange of the exocyclic sulfur for oxygen to give 1,3-oxathiolan-2-one **2** (Scheme 1). We therefore sought to optimize this transformation.

The starting thione **1** is readily available, having first been prepared at a very early date by reaction of sodium trithiocarbonate with 1,2-dibromoethane (Husemann, 1862). Later and more convenient preparations include reaction of 1,2-dichloroethane with carbon disulfide in the presence of a base (Coltof, 1940) or with sodium trithiocarbonate under phase-transfer conditions (Degani et al., 1986) and, most convenient and economical on an industrial scale, base-induced reaction of carbon disulfide and ethylene oxide (Culvenor et al., 1946). The product **2** is also well known and, after some initial confusion with the isomeric 1,3-oxathiolane-2-thione structure (Husemann, 1863), its identity was firmly established by the end of the 19th century (Miolati, 1891; Busch and Lingenbrink, 1900). Most significantly for the current project, the conversion of **1** into **2** has been achieved using a variety of reagents but most of these are highly toxic or hazardous and lead to formation of harmful waste. A clean method using air as the ultimate oxidant would therefore be highly desirable and fulfill many of the requirements for a “green chemistry” process. By way of contrast, the existing methods include stoichiometric use of mercuric acetate (Challenger et al., 1953), mercuric oxide/acetic anhydride (Overberger and Bonsignore, 1958), polymer-supported selenoxide and telluroxide reagents (Hu et al., 1986), dimethyl sulfate followed by acid hydrolysis (Degani et al., 1994), epoxycyclohexane (Barbero et al., 1996) and potassium permanganate under phase transfer conditions (Aitken et al., 1997c). Compound **2** is long known to be a convenient precursor for synthesis of the useful unsaturated heterocycles **3** and **4** (Mayer and Gebhardt, 1964), and there have been various recent applications of the product **2** including as a component of the electrolyte in high-temperature batteries (Ihara et al., 2008), as a key intermediate in the chemical recycling of waste polycarbonate (Hata et al., 2003), and as a precursor for binuclear iron-sulfur carbonyl complexes (Lagadec et al., 1987; Xiao et al., 2017).

## Results and Discussion

Molybdenum trixode is a readily available and thermodynamically stable material that has found use as a catalyst in many large-scale catalytic processes (Sebenik et al., 2012). Significantly for what follows, it is manufactured by roasting the ore molybdenite (MoS_2_) in air at 600°C. For our studies, we used pumice stone as an inert support material. This is a readily available mineral whose surface properties have been studied in detail (Brito et al., 2004) and which has recently found use as a catalyst support for oxidative waste water treatment (Alver and Kilic, 2018). The solid reagent for our studies was prepared either by directly shaking a mixture of commercially available pumice stone chips (BET surface area 2.2 m^2^g^−1^) with finely powdered MoO_3_ or by the solution impregnation method which has been commonly used to prepare supported MoO_3_ catalysts (Hu and Wachs, 1995; Chary et al., 2004; Rathod et al., 2014). In this the pumice stone chips were stirred in an aqueous solution of ammonium molybdate at 80°C for 5 h. The mixture was then evaporated to dryness and the residual solid first dried at 100°C and then calcined at 500°C in air.

Initial studies quickly established that for effective reaction, the molybdenum trioxide should be evenly dispersed on a large amount of the solid support which filled the reaction tube (Table 1). For example while a packing of 1.25 g MoO_3_ on 5 g pumice gave only 11% conversion, this was raised to 96% using a packing of 0.77 g MoO_3_ on 50 g pumice (runs 1 and 2). Varying the temperature (runs 3–6) showed that for high conversion a temperature of at least 450–500°C was required. However, when a larger scale run was carried out at 500°C a problem became apparent as, despite a high degree of conversion, only 15% isolated yield was obtained. This was due to decomposition of product **2** under the conditions used, something that was readily confirmed by passing **2** through pumice at 450°C and 10^−2^ Torr which resulted in only 24% recovery with 76% decomposition into gases and other volatile products such as thiirane **5** which was detected by NMR in some runs. Therefore, a slightly lower reaction temperature may be preferable as the lower conversion is more than made up for by the lower extent of product decomposition leading to a higher isolated yield.

**Table 1:** Results for gas-phase conversion of 1,3-dithiole-2-thione into 1,3-dithiol-2-one over molybdenum trioxide.

**Run**	**Quantity of 1 (mg)**	**Temperature (**°**C)**	**Pressure (Torr)**	**Reagent preparation**	**Starting material %**	**Product 2 %**	**Isolated yield %**
1	40	550	10^−2^	A	89	11	—
2	32	550	10^−2^	B	4	96	—
3	42	350	10^−2^	B	64	36	—
4	35	400	10^−2^	B	35	65	—
5	35	450	10^−2^	B	19	81	—
6	43	500	10^−2^	B	8	92	—
7	498	500	10^−2^	B	12	88	15
8^*^	500	500	10^−2^	B	80	20	—
9	40	500	10^−2^	C	4	96	8
10	40	450	10^−2^	C	9	91	62
11	30	400	10^−2^	C	28	72	67
12	40	300	10^−2^	C	32	68	39
13	77	400	10^−2^	D	0.4	99.6	42
14	2020	400	760	D	40	60	19
15	52	250	760	C	59	41	15
16	34	300	760	C	31	69	53
17	46	350	760	C	24	76	12

*Reagent preparation: A = MoO_*3*_ (1.25 g) shaken with pumice (5 g); B = MoO_*3*_ (0.77 g) shaken with pumice (50 g); C = pumice (50 g) impregnated with MoO_*3*_ from ammonium molybdate (0.91 g); D = pumice (50 g) impregnated with MoO_*3*_ from ammonium molybdate (9.1 g)*.

* *Run conducted immediately after previous one with no exposure of reagent to air in between*.

**Scheme 2:**
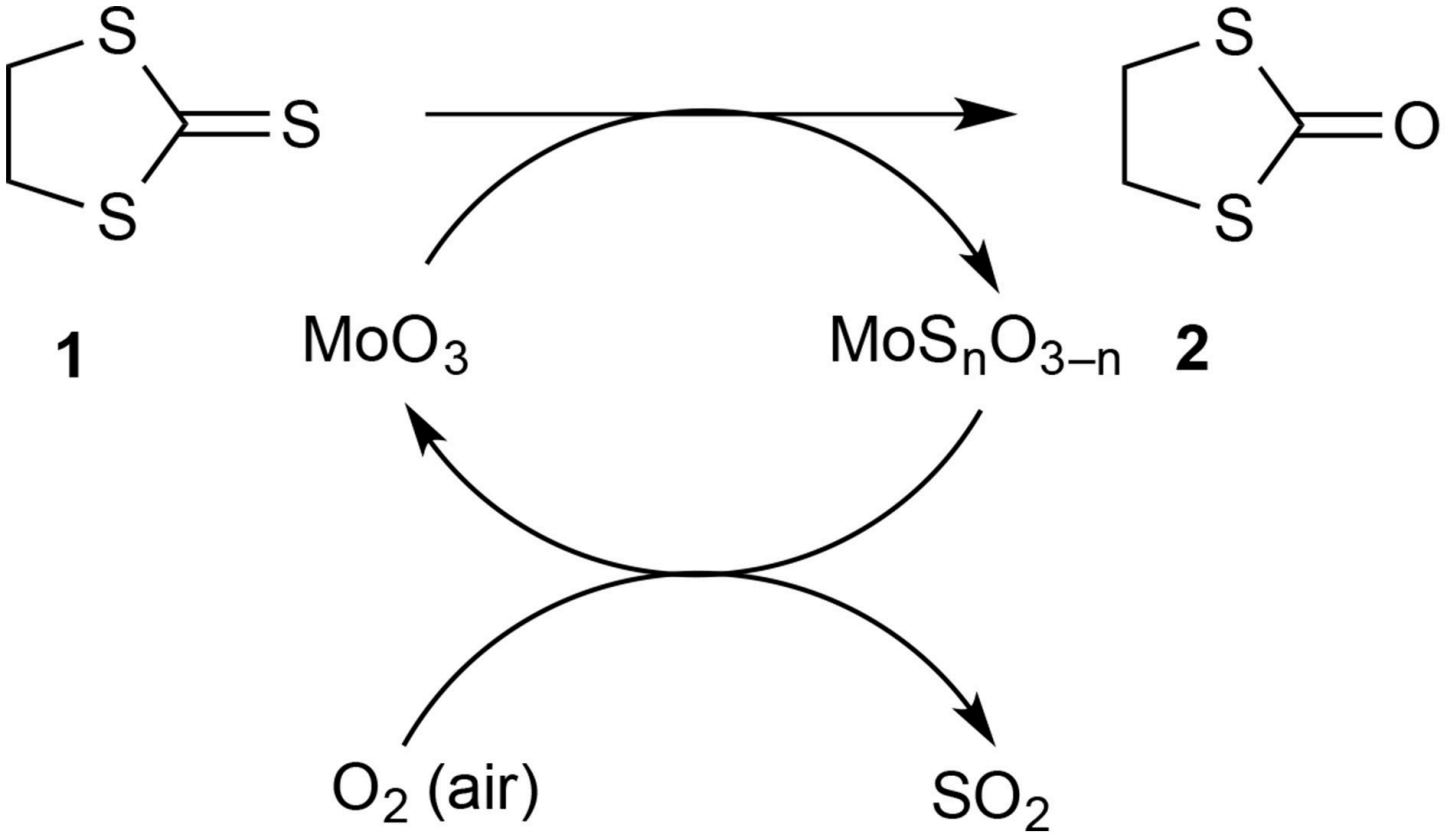
Conversion of **1** into **2** with air regeneration of the catalyst.

We were keen to evaluate the capacity of our solid-supported reagent and estimate how much of the MoO_3_ was available for reaction. For this, the same tube of reagent was used repeatedly in the hope of exhausting the reagent and eventually observing a drop in conversion. However, this did not happen and this led to the realization that the reagent was being regenerated by exposure to air between runs. This was easily demonstrated by conducting successive runs on a 500 mg scale and leaving the hot reagent bed exposed to air for 30 min between runs giving 76 then 77% conversion. However, when a run was done as quickly as possible after the previous one with minimal exposure of the reagent to air in between, a drop in conversion to 20% was observed (run 8). A conventional run after this with exposure of the reagent to air saw a return to 74% conversion. A useful qualitative indicator for the state of the reagent bed was its color: the initially pure white solid became bright greenish-yellow upon partial sulfurization and then returned to white after regeneration in air. The fact that the partly sulfurized MoO_3_ is reconverted to MoO_3_ on exposure to air at 500°C should come as no surprise since this is essentially the process by which it is manufactured from molybdenite ore (MoS_2_). This has the important consequence that the process for converting **1** into **2** can in principle be catalytic in MoO_3_ with oxygen of the air as the ultimate oxidant and the eliminated sulfur ending up as SO_2_ which could be readily trapped from the gas phase if desired (Scheme 2).

We now turned to the solution impregnation method to prepare the solid reagent/catalyst and as expected this gave significantly better results. Predictably, decreasing the reaction temperature from 500°C to 300°C resulted in progressively lower conversion (runs 9–12), but this was accompanied by less product decomposition leading to higher isolated yields particularly at 400°C which at 67% gave the highest isolated yield in the current study. A second reagent sample prepared by the impregnation method but using ten times more ammonium molybdate gave excellent conversion at 400°C (run 13) but unfortunately this was accompanied by significant decomposition leaving an isolated yield of only 42%. When this high molybdenum catalyst was used for a large-scale preparation at atmospheric pressure, both the conversion and isolated yield were poorer and slight traces of both the dehydrogenation products 1,3-dithiole-2-thione **3** and 1,3-dithiol-2-one **4** were evident (run 14).

Since we were well aware that the requirement for high vacuum is problematic for scaling up any flow process, we decided to examine this reaction under atmospheric pressure flow conditions. Using a stream of nitrogen as carrier gas and the solid catalyst prepared by impregnation, the conversion and isolated yield were found to be optimal at a furnace temperature of 300°C with 53% isolated yield and poorer results were obtained at either 250°C or 350°C (runs 15–17).

In conclusion, we have demonstrated that molybdenum trioxide supported on pumice stone is an effective reagent for the gas-phase conversion of dithiolanethione **1** into the corresponding dithiolanone **2** under both FVP and atmospheric pressure flow conditions. To maximize the isolated product yield it may be desirable in each case to operate at somewhat below the temperature of maximum conversion so that product loss by decomposition is minimized. As a bonus, the supported reagent is fully regenerated on exposure to air and may thus act as a catalyst with overall consumption of oxygen from the air and production of sulfur dioxide in the course of converting **1** into **2**. Further applications of gas-phase reactions over supported molybdenum trioxide in heterocyclic chemistry will be reported shortly.

## Experimental

### Preparation of Chemicals

1,3-dithiolane-2-thione **1** was prepared by the literature method (Culvenor et al., 1946) involving reaction of ethylene oxide with carbon disulfide and potassium hydroxide in methanol and was obtained as yellow crystals, mp 36–37°C. ^1^H NMR (CDCl_3_) δ = 4.00 (s); ^13^C NMR (CDCl_3_) δ = 228.8 (C = S), 43.7 (CH_2_).

1,3-dithiolan-2-one **2** for reference was prepared by the literature method (Aitken et al., 1997c) involving KMnO_4_ oxidation of **1** under phase transfer conditions and was obtained as pale yellow crystals, mp 35–36°C. ^1^H NMR (CDCl_3_) δ = 3.72 (s); ^13^C NMR (CDCl_3_) δ = 198.5 (C = O), 36.2 (CH_2_).

### Pyrolysis Equipment and Reaction Procedure

Gas phase reactions were carried out using a horizontal quartz reaction tube (30 × 2.5 cm) heated in a Carbolite Eurotherm laboratory tube furnace MTF-12/38A, the temperature being measured by a pt/pt-13% Rh thermocouple located at the center of the furnace. The reaction tube was packed with the solid reagent (50 g) sandwiched between plugs of glass wool. For vacuum experiments the system was connected via a *U*-shaped cold trap to an Edwards model E2M5 high capacity rotary oil pump giving pressures of 10^−3^–10^−2^ Torr and the starting material was volatilized by external heating of the inlet tube. For atmospheric pressure experiments the starting material was volatilized by external heating of the inlet tube and carried through the reaction tube by a slow flow of nitrogen gas to a U-shaped cold trap.

### Preparation of Solid Reagent/Catalyst

Finely powdered molybdenum (VI) oxide was obtained from Acros Organics and granular pumice stone was obtained from Fluka with grain size 4–6 mm. The BET surface area of the latter as determined by porosimetry was 2.2 m^2^g^−1^. The preparation of the supported reagent/catalyst was done in two separate ways. In the first, the finely powdered MoO_3_ (0.77 g) was shaken in a bottle with pumice stone chips (50 g) until the two solids were intimately mixed and the pumice was evenly coated with MoO_3_. In the second method, ammonium molybdate tetrahydrate (0.91 g) was stirred with pumice stone chips (50 g) in water (250 mL) at 80°C for 5 h. The mixture was then evaporated using a rotary evaporator and the resulting solid was first dried in an oven at 100°C for 16 h and then calcined by heating in the reaction tube at 500°C under slight vacuum for 5 h. Before use the reaction tube packed with solid reagent/catalyst was heated to 500°C open to the air for 30 min to drive off adsorbed moisture.

### Analysis of Products

This was performed using NMR. The product was dissolved out from the cold trap using CDCl_3_ and the ratio of starting material **1** to product **2** was determined by integration of the ^1^H NMR signals at δ 4.00 and 3.72, respectively. Confirmation of the identity of the products was obtained by ^13^C NMR and comparison with the literature data (Poleschner et al., 1981). Typical ^1^H and ^13^C NMR spectra are shown in the Supplementary Material and trace components also evident there (Table 1, run 14) are 1,3-dithiole-2-thione **3** (δ_H_ 7.18, δ_C_ 129.3) and 1,3-dithiol-2-one **4** (δ_H_ 6.83, δ_C_ 118.2). In some experiments thiirane **5** (δ_H_ 2.39, δ_C_ 18.1) was also observed.

## Author Contributions

TC and MA carried out the experimental work and processed the results. RA conceived the study, analyzed and interpreted the results and wrote the paper.

### Conflict of Interest Statement

The authors declare that the research was conducted in the absence of any commercial or financial relationships that could be construed as a potential conflict of interest.
