# Supplementary material for: Gas-Phase Conversion of 1,3-Dithiolane-2-Thione Into 1,3-Dithiolan-2-One Over Molybdenum Trioxide
Source: Front Chem. 2019 Apr 5;7:204. doi: 10.3389/fchem.2019.00204 (PMC6460114; doi:10.3389/fchem.2019.00204)

Supplementary Material

Gas-phase Conversion of 1,3-Dithiolane-2-thione into 1,3-Dithiolan-2-one over Molybdenum Trioxide

R. Alan Aitken*, Thomasine E. Curzon and Matthew J. Andrews

EaStCHEM School of Chemistry, University of St Andrews, St Andrews, Fife, UK

*** Correspondence:**
Dr R. Alan Aitken
raa@st-and.ac.uk

Figure S1: 300 MHz ^{1}H NMR spectrum of typical product mixture (run 14)

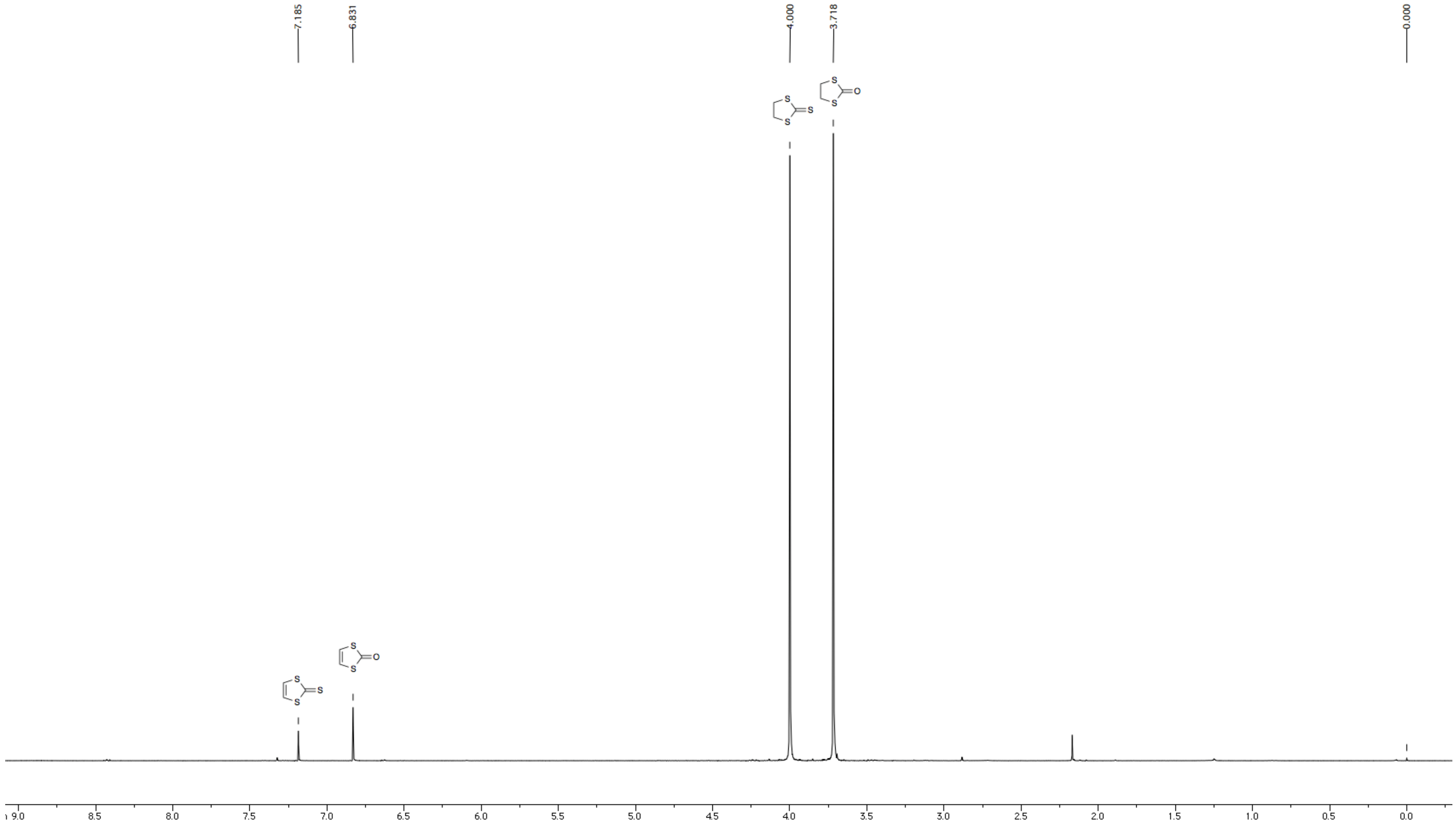

Figure S2: 75 MHz ^{13}C DEPTQ NMR spectrum of typical product mixture (run 14)

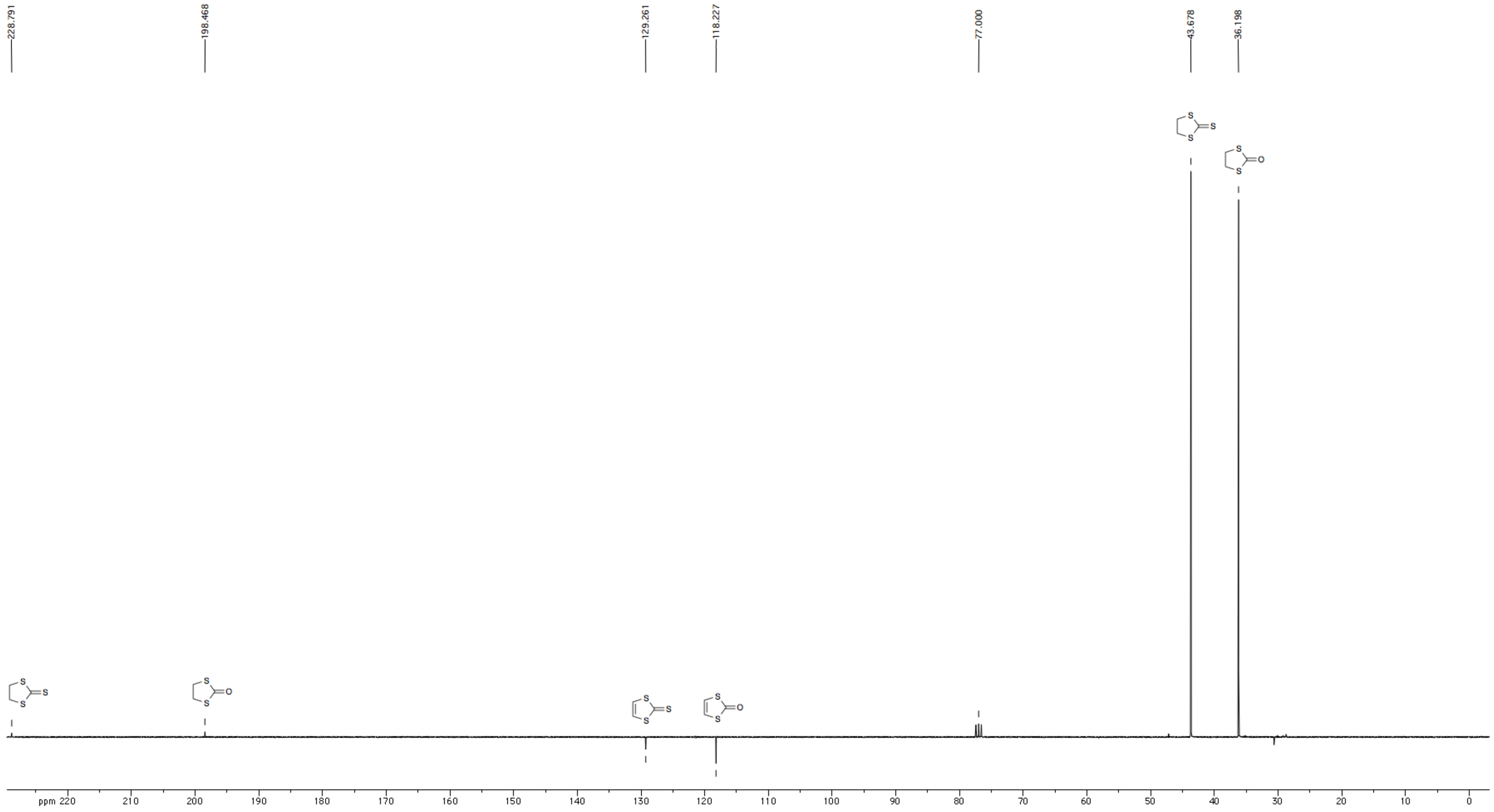

Supplement: Supplementary file 1 [file Presentation_1.pdf]
